# Perceptions of Dentists and Non-Professionals on Some Dental Factors Affecting Smile Aesthetics: A Study from Vietnam

**DOI:** 10.3390/ijerph17051638

**Published:** 2020-03-03

**Authors:** Vo Truong Nhu Ngoc, Dang-Khoa Tran, Truong Manh Dung, Nguyen Viet Anh, Vu Thi Nga, Le Quynh Anh, Nguyen Thi Thuy Hanh, Nguyen Phuong Linh, Hoang Ngoc Quynh, Dinh Toi Chu

**Affiliations:** 1 School of Odonto Stomatology, Hanoi Medical University, Hanoi 100000, Vietnam; nhungoc@hmu.edu.vn (V.T.N.N.); Dzungvrhm@yahoo.co.uk (T.M.D.); lequynhanh@hmu.edu.vn (L.Q.A.); 2 Department of Anatomy, University of Medicine Pham Ngoc Thach, Ho Chi Minh City 700000, Vietnam; khoatrandr@gmail.com; 3 Institute for Research and Development, Duy Tan University, Danang 550000, Vietnam; 4 Sydney Dental School, Faculty of Health and Medicine, The University of Sydney, Science Rd, Camperdown NSW 2050, Australia; 5 Institute for Preventive medicine and Public Health, Hanoi Medical University, Hanoi 100000, Vietnam; nguyenthuyhanh@hmu.edu.vn; 6 Ban Mai School, Ha Dong, Hanoi 100000, Vietnam; linhphuongnguyen93@gmail.com; 7 Nottingham Trent University, 50 Shakespeare St, Nottingham NG1 4FQ, UK; n.q.hoang@lancaster.ac.uk; 8 Faculty of Biology, Hanoi National University of Education, Hanoi 100000, Vietnam

**Keywords:** smile aesthetics, perception, dentists and non-professionals

## Abstract

*Aim*: It is important to meet the aesthetic expectation regarding the smile concept of both dentists and non-professionals after treatment is complete. Therefore, the study aims to evaluate the effects of altered displays in incisors, gingival margin, and other smile-related-factors on dentists’ vs. non-professionals’ aesthetics perceptions. *Materials and method*: We altered the features of 42 digital smile photographs to generate the changed displays in incisors, gingival margin, and other smile-related-factors. Then, these altered photographs were presented to 51 dentists and 51 non-professionals, and each picture was rated by each participant with a visual analog scale ranging from 0 (very ugly) to 100 (very beautiful). *Results*: We found that the alterations in incisors, gingival margin, and other factors affected studied groups’ aesthetic perception of smile. The ugly smile threshold rated by both groups for crown length of maxillary central incisors was 2.0 mm. This threshold was 2.5 mm for dentists, with moving the gingival margin of maxillary lateral incisors to the incisal ridge. The ugly thresholds for other smile-related-factors were different between studied groups; for example, the ugly thresholds for gingival exposure levels were 3 and 4mm for dentists and non-professionals, respectively. Thus, our data indicate that altered displays in incisors, gingival margin, and other smile-related-factors affected perceptions of both studied groups on smile aesthetics, but dentists tended to feel more refined than non-professionals. Dentists and non-professionals had significantly different aesthetic perceptions of the alteration of the gingival exposure level. *Conclusion*: Both dentists and non-professionals’ perceptions should be fully considered during orthodontic and prosthodontic treatment to achieve optimum aesthetic results.

## 1. Introduction

With the social development and enhanced quality of life, the human need for aesthetics has been increasingly growing. As smile is one of the factors that strongly affect facial aesthetics, the field of smile aesthetics has been focusing highly on dental specialties, including orthodontics, prosthodontics, periodontics, and oral-facial surgery. The concepts of smile attractiveness and smile design have become popular in dentistry. Smile attractiveness is strongly affected by several factors, such as the shape, size, and color of the teeth, teeth arrangement, the space between the teeth, and the surrounding structures (lips and gums) [1,2]. Occlusal cant and gingival display have also been proved to have a strong impact on smile aesthetics [3,4].

However, many patients, with a single or multiple reasons after finishing orthodontic and prosthodontic treatment clinically, cannot achieve ideal aesthetic results. Some factors have not reached a certain benchmark, for example, dental midline shift or inappropriate length/width ratio of crown, lateral incisor too small, midline diastema, gingival margin differences in anterior teeth, as well as gummy smile or occlusal plane discrepancies. Thus, in order to achieve desirable aesthetic smile outcomes and to satisfy patients, dentists need to know the acceptable standard deviation within this interval.

In addition, smile aesthetics are strongly affected by the subjective perception of each individual. Dentists, who often have to deal with aesthetic issues, may be more subtle than non-professionals [5,6]. However, the non-professionals are the aesthetic customers who pay for dental specialties. The question is whether or not the allowed deviations of the above factors are different between dentists and non-professionals.

Therefore, it is essential to determine the allowed deviation of the factors related to smile aesthetics for clinical dentistry. However, there has been no research done in this area in Vietnam. Herein, we report the study with the primary objective of identifying the required modification level in several aesthetic factors related to the smile, which were assessed by both groups. The similarities and differences in perceptions of beautiful smiles among professional and non-professional groups suggest important criteria to optimize the most satisfactory aesthetic results.

## 2. Materials and Methods

### 2.1. Materials

Two assessment groups included 51 dentists and 51 non-professionals. The group of dentists consisted of postgraduate students at the School of Odonto-Stomatology, Hanoi Medical University. The group of non-professionals was students of National Economics University, Hanoi, Vietnam.

The smile images were generated using graphical software to adjust seven original snapshots. These photographs were taken from the project at the national level: Research on characteristics of orofacial anthropometry in Vietnamese for medical appliance.

#### Selective Criteria

Dentists: Doctors who have been graduated as Doctor of Dental Surgery or Dental-Oriented Specialty Degree for more than 2 years.

Non-professionals: Those who are not working in the dental industry or dental-related work.

Forty-two smile images were made up from 7 original snapshots of 7 different people. The selected smile must be relatively and aesthetically beautiful in manner: aligned teeth, proper correlation between the gingival margin and incisal ridge, less than 2mm gingival exposure, no tilted occlusal plane, and no diastema.

### 2.2. Method

Cross sectional study on 102 subjects, including 51 dentists and 51 non-professionals.

#### Study Procedure

Seven standardized smile images of seven different people were selected by capturing only the lips and the teeth and eliminating the nose and chin to avoid bias. Adobe Photoshop CS6 was used to adjust the following factors: the width of maxillary lateral incisors, the length of maxillary central incisors, gingival margin of maxillary lateral incisors, gingival exposure, maxillary midline diastema, maxillary midline shift, and tilted occlusal plane. A total of 42 photos were produced following the above adjustment criteria.

**Figure 1:**
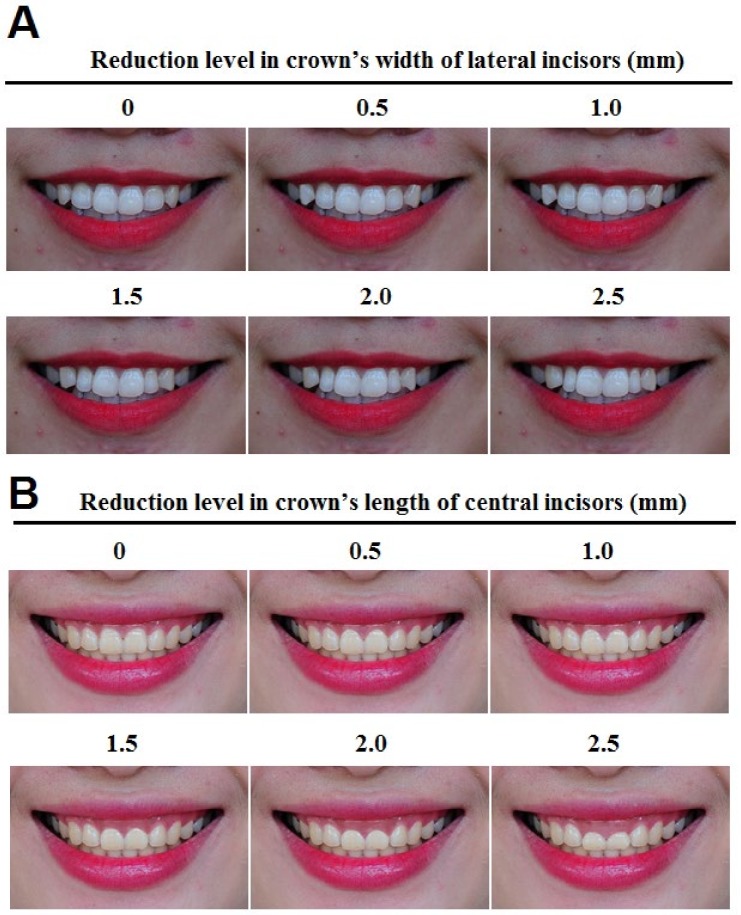
Modification on the crown’s width of maxillary lateral incisors (**A**), and on the crown’s length of maxillary centrals (**B**).

*The crown width of the maxillary lateral incisors*: The crown width of these teeth was reduced. The latter photo was 0.5 mm narrower than the former one while keeping the incisal ridge position (Figure 1A).

*The crown length of maxillary central incisors*: The crown length of these incisors was shortened; the latter photo was 0.5 mm shorter than the former one by lowering the gingival margin but still keeping the incisal ridge position (Figure 1B).

**Figure 2:**
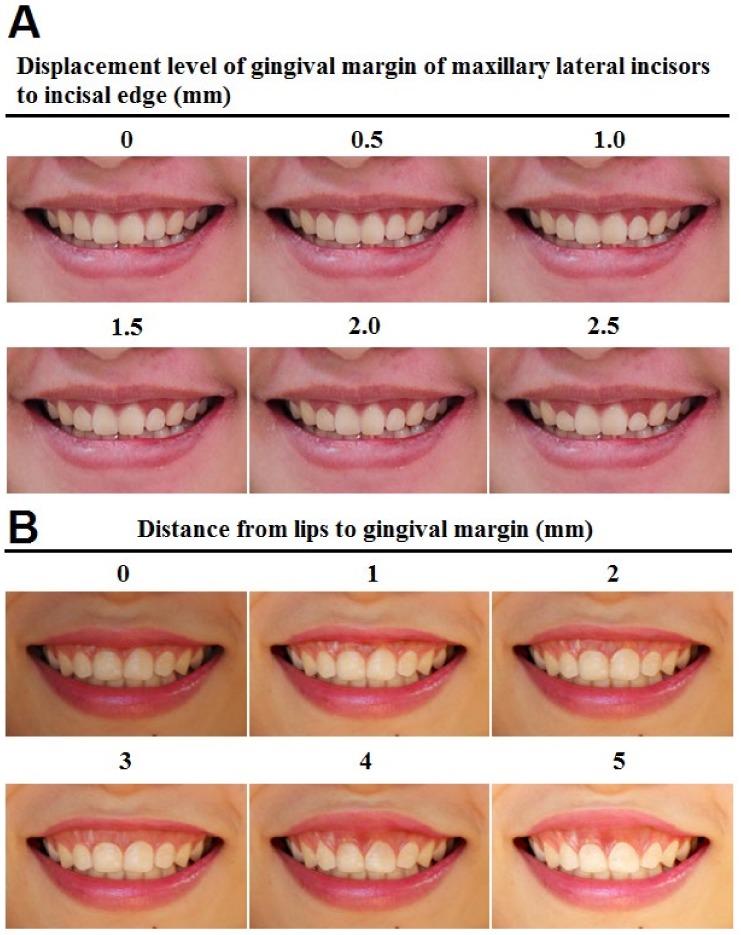
Modification on gingival margin position of maxillary lateral incisors (**A**), and on gingival exposure level (**B**).

*The gingival margin position of maxillary lateral incisors*: The margin position of these teeth was moved towards the incisal ridge. The latter photo was 0.5mm lower than the previous one, while the gingival margin of the maxillary central incisors and maxillary canine were maintained (Figure 2A).

*Gingival exposure level*: Gingival exposure was measured from the upper lip to the gingival margin of the maxillary central incisors. The photos were adjusted by moving the upper lip to have display levels of 0.5, 1, 2, 3, 4, and 5mm, respectively (Figure 2B).

**Figure 3:**
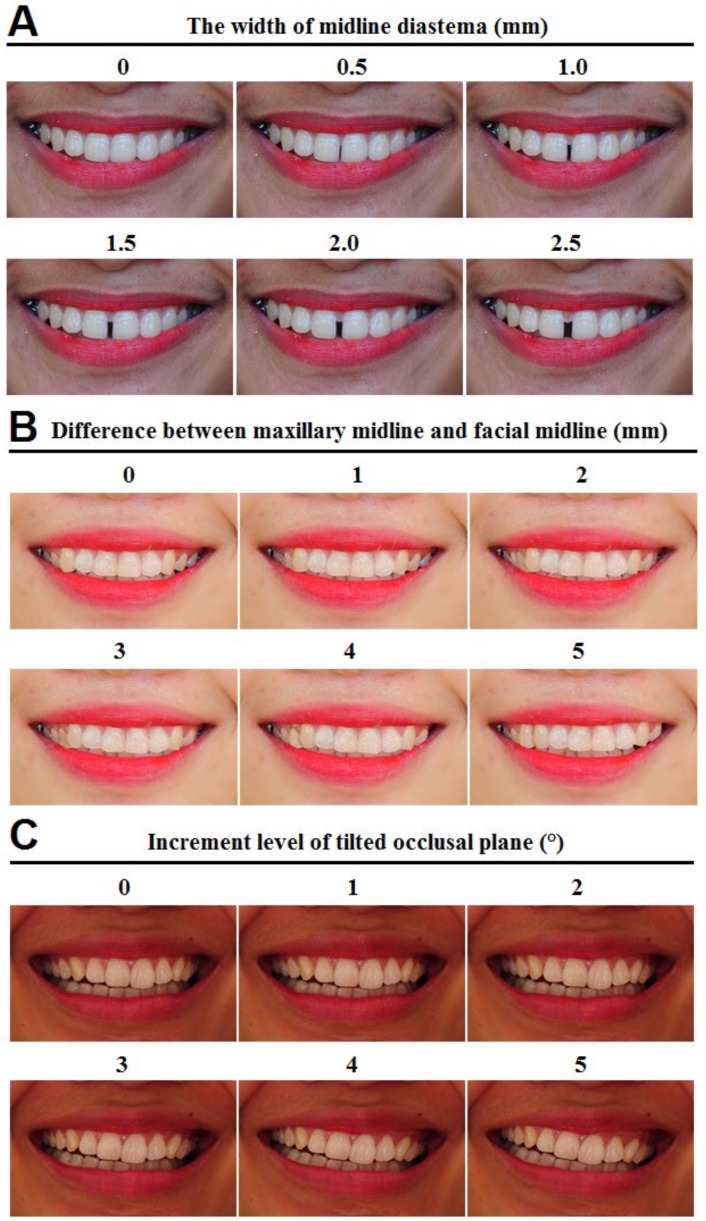
Maxillary midline diastema (**A**), maxillary midline shift (**B**), and tilted occlusal plane (**C**).

*Maxillary midline diastema*: Maxillary midline diastema, which was measured by distance between interproximal contacts of two maxillary central incisors. This gap was adjusted to be 0.5mm wider in each photo (Figure 3A).

*Maxillary midline shift*: Maxillary midline shift was adjusted to the left side gradually with the increase of 1mm in each photo, while the mandible midline and Cupid’s bow were fixed reference points (Figure 3B).

*Tilted occlusal plane*: Occlusal plane was gradually rotated clockwise by 1° in each photo. Both lips were fixed as reference points (Figure 3C).

*Assessment scale*: Each subject in the two research groups of dentists and non-professionals evaluated each image, and below each one is a 100mm ruler for aesthetic assessment. The closer to 100mm the subject evaluated the photo, the more beautiful the smile was. The closer to 0mm that the subject evaluated the photo, the uglier the smile was. In detail: 0–20 mm (very ugly), 20–40 mm (ugly), 40–60 mm (moderate), 60–80 mm (beautiful), 80–100 mm (very beautiful). Photos were arranged randomly for each subject. Each subject had 10 s to grade each image.

### 2.3. Research Ethics

This study was conducted only with consent forms for using available images signed by the research subjects and the head of the national level project. All procedures performed in the studies involving human participants were in accordance with the ethical standards of the School of Odonto-Stomatology’s Ethical Committee, Hanoi Medical University, and with the 1964 Helsinki declaration and its later amendments or comparable ethical standards.

### 2.4. Statistical Analysis

The SPSS 16.0 software (IBM, Armonk, NY, USA) was employed to calculate the average point (x) and standard deviation (SD) of each study group for each image as reported previously [7]. Statistically significant differences in perceptions between dentists and non-professionals for each image, which were determined by Student’s *t*-test with *p* < 0.05 were considered as significant.

## 3. Results

### 3.1. The Effect of Alterations of the Crown Width/Length of the Maxillary Lateral/Central Incisors on Aesthetic Perceptions between Studied Groups

The maximum reductions in the crown width of maxillary lateral incisors (Figure 1A) that non-professionals and dentists still considered it a beautiful smile were 2.0 mm and 0.5 mm, respectively. Interestingly, dentists scored more rigorously than non-professionals when decreasing the crown width of maxillary lateral incisors, and the difference was statistically significant when the reduction was 2.5 mm (*p* = 0.008) (Table 1), although both groups did not perceive this smile to be ugly.

However, when the crown length of the maxillary central incisors was decreased by 2.0 mm or more (Figure 1B), both non-professionals and dentists rated the smile as ugly. And we did not see any statistically significant difference between dentists’ and non-professionals’ perceptions of these incisors’ reduced crown length. (Table 1).

### 3.2. The Effect of Alterations of the Gingival Margin Position of Maxillary Lateral Incisors or Gingival Exposure Level on Aesthetic Perceptions between Studied Groups

For the gingival margin position (Figure 2A), when we displaced 2.5 mm of the gingival margin of the maxillary lateral incisors to the incisal edge, the group of dentists rated the smile as ugly while the other group considered it a moderate smile (Table 2). The dentists rated the smile aesthetics more rigorously than the non-professionals with a displacement level of 1.0mm or more, but the difference was not statistically significant (Table 2).

In regard to gingival exposure level (Figure 2B), non-professionals and dentists perceived the smile to be ugly when exposure levels were 4 and 3 mm, respectively (Table 2). The dentist group assessed smile aesthetics more strictly than the other group when the exposure level was 1 mm or more. There were statistically significant differences when exposure levels were 2, 3, and 5 mm (Table 2).

### 3.3. The Effect of Alterations of the Maxillary Midline Diastema, Maxillary Midline Shift, and Tilted Occlusal Plane on Aesthetic Perceptions between Studied Groups

For maxillary midline diastema (Figure 3A), the width of the maxillary midline diastema with which the non-professionals and the dentists felt that the smile is ugly were 1.0 and 1.5mm, respectively. However, no significant difference between the two studied groups was found when we changed the width of the maxillary midline diastema (Table 3).

For maxillary midline shift (Figure 3B), when the maxillary midline was shifted 5mm, both groups did not evaluate the smile as ugly. The perception of the dentist group shifted more dramatically than that of non-professionals with increasing the deviation of the midline, but the difference was not statistically significant (Table 3).

For tilted occlusal plane (Figure 3C), non-professionals and dentists perceived a smile to be ugly when the occlusal plane was tilted 5° and 4° and above, respectively. The group of dentists rated more rigorously when the occlusal plane was tilted 2° or more; however, no significant differences were observed (Table 4).

## 4. Discussion

In this study, dentists and non-professionals considered a smile ugly when the reduction in crown length was ≥2 mm. This finding is quite consistent with the result in the previous study [8], in which Kokich et al. found that the thresholds of ugly smiles were 1.5 and 2.0 mm for general dentists and non-professionals, respectively. The crown length of the upper incisors must be greater than the crown width. Usually, the length/width ratio is 1:0.8 [9], which means that the incisors’ shape must be rectangular rather than square. For patients with short crowns, this can be improved by composite restorations, porcelain restorations, or gingivectomy.

According to Kokich et al. [8], dentists and non-professionals felt that a smile was ugly when the crown width of maxillary lateral incisors was reduced by 3 and 4 mm, respectively. This result is quite similar to our research findings, in which we found that both dentists and non-professionals did not consider a smile ugly with a reduction of 2.5 mm in the crown width of these teeth. In order to have an aesthetic smile, the crown width ratio (maxillary lateral incisor:maxillary central incisor) should be 0.618:1 (golden ratio). If the patient has wedge-shaped lateral incisors, orthodontic treatment may be necessary to create enough space, then prepare composite or porcelain restoration to achieve a golden ratio.

Our data showed that when moving the gingival margin of maxillary lateral incisors to the incisal ridge 2.5 mm, the dentists evaluated the smiles as ugly, while the non-professionals did not feel the same way. This finding is consistent with the study done by Kokich et al. [8], in which, by displacing 2 mm, both dentists and non-professionals did not perceive the smile as ugly.

The study results also showed that the minimum gingival disclosure levels in which non-professionals and dentists felt a smile ugly were 4 and 3 mm, respectively. This finding is considerably similar to the result reported by Kokich et al. [8], where the gingival exposure threshold for both general dentists and non-professionals to consider a smile ugly was 4 mm.

From the above results, dentists and non-professionals felt a smile ugly when the minimum width of maxillary midline diastema was 1.5 and 1.0 mm, respectively. This result is similar to that of Nabeel et al. [5], showing that the thresholds for ugly smile perception of dentists and non-professionals were 1.0 and 1.5 mm, respectively.

Our study also found that when the midline shift was 5 mm, both dentists and non-professionals did not rate the smile as poorly aesthetic. This finding is consistent with the study reported by Kokich et al. [8], in which both general dentists and non-professionals failed to detect a 4 mm difference in the midline.

In addition, the results showed that when the plane was tilted 5°, the non-professional group began to evaluate the smile as ugly. Meanwhile, the dentist group perceived the smile ugly at a 4° tilted occlusal plane. This result is perfectly consistent with two other studies. Research by Ker et al. [10] pointed out that the threshold for tilting the occlusal plane was 4° for the non-professionals, while Silva et al. [11] found that non-professionals began to feel the smiles as poorly aesthetic at a 5° tilted occlusal plane.

Finally, no difference between the two groups was found when the crown length of maxillary central incisors was decreased and the crown width of maxillary midline diastema was increased. There was also no statistically significant difference between the studied groups regarding the following factors: the crown width of the maxillary lateral incisors, gingival margin position of maxillary lateral incisors, maxillary midline diastema, and occlusal plane discrepancies. However, dentists and the non-professionals had different perceptions when we increased the gingival exposure level.

Deviations of some aesthetic factors in relation to professional dentists and laypersons have been studied in the world, such as in the United States by Kokich et al. [8] and by Ker et al. [10]. However, to our knowledge, there has been no similar study done in Vietnamese people, while it is very important to meet the aesthetic expectation regarding the smile concept of both dentists and non-professionals after treatment is complete. Thus, this research has determined and shared the findings in the allowed deviations of some aesthetic factors in relation to professional dentists and laypersons in Vietnam.

## 5. Conclusions

The threshold of ugly smile perception with the factors of reducing the crown length of maxillary central incisors: dentists (2.0 mm), non-professionals (2.0 mm); moving the gingival margin of maxillary lateral incisors to the incisal ridge: dentists (2.5 mm); gingival disclosure level: dentists (3 mm), non-professionals (4 mm); maxillary midline diastema: dentists (1.5 mm), non-professionals (1.0 mm); tilted occlusal plane: dentists (4°), non-professionals (5°). By either decreasing the crown width of the maxillary lateral incisors or shifting midline, at maximum deviation in this study (2.5 and 5 mm), both studied groups did not perceive the smiles as ugly.

From our results, we conclude that when assessing some effects on the smile aesthetics of Vietnamese people, dentists tend to feel more refined than non-professionals. They had different perceptions of smile aesthetics when the gingival exposure level was changed. Therefore, it is in need of consideration during orthodontic and prosthodontic treatment to achieve optimum aesthetic results.

## Figures and Tables

**Table 1:** Perceptions of dentists and non-professionals towards some maxillary incisors—related factors.

Maxillary Incisors—Related Factors	Non-Professionals		Dentists		*p*
	x	SD	x	SD	
**Reduction level in crown’s width of lateral incisors (mm)**					
**0**	69.37	17.68	65.07	15.50	0.195
**0.5**	65.76	17.75	63.72	13.89	0.520
**1.0**	64.27	17.85	58.47	12.87	0.063
**1.5**	65.59	17.44	59.98	15.62	0.090
**2.0**	60.39	15.64	54.80	12.75	0.051
**2.5**	55.78	16.89	46.62	17.09	0.008 *
**Reduction level in crown’s length of central incisors (mm)**					
**0**	59.39	15.07	59.76	14.32	0.898
**0.5**	52.25	17.89	54.29	15.69	0.542
**1.0**	52.88	15.82	49.50	16.36	0.293
**1.5**	44.35	15.88	42.11	14.11	0.454
**2.0**	31.96	15.46	33.41	14.31	0.624
**2.5**	26.50	15.30	23.92	13.74	0.371

* Significantly different.

**Table 2:** Perceptions of dentists and non-professionals towards the gingival margin position.

Gingival Margin Position	Non-Professionals		Dentists		*p*
	x	SD	x	SD	
**Displacement level of gingival margin of maxillary lateral incisors to incisal edge (mm)**					
**0**	56.94	16.15	58.07	13.55	0.701
**0.5**	60.49	14.02	60.68	13.48	0.943
**1.0**	57.50	15.86	54.64	14.57	0.345
**1.5**	52.92	15.20	50.98	14.25	0.508
**2.0**	55.33	12.95	53.66	14.88	0.548
**2.5**	41.07	18.12	39.25	17.23	0.604
**Distance from lips to gingival margin (mm)**					
**0**	61.51	15.43	61.90	14.37	0.895
**1**	51.53	16.20	48.92	12.03	0.358
**2**	46.67	16.81	40.43	10.09	0.025 *
**3**	43.61	18.68	35.71	12.25	0.013 *
**4**	39.00	16.24	34.49	14.00	0.136
**5**	36.35	15.64	29.65	13.48	0.022 *

* Significantly different.

**Table 3:** Perceptions of dentists and non-professionals towards some midline factors.

Midline Factors	Non-Professionals		Dentists		*p*
	x	SD	x	SD	
**The width of midline diastema (mm)**					
**0**	61.53	18.48	56.57	17.48	0.167
**0.5**	51.02	16.90	48.41	15.46	0.418
**1.0**	37.71	16.15	40.96	14.48	0.287
**1.5**	32.24	14.38	33.88	13.84	0.557
**2.0**	30.29	13.17	32.67	11.96	0.343
**2.5**	25.80	12.74	25.92	12.19	0.962
**Difference between maxillary midline and facial midline (mm)**					
**0**	61.98	15.51	58.18	17.38	0.246
**1**	59.96	15.68	58.27	14.32	0.572
**2**	58.02	16.96	54.18	15.62	0.237
**3**	59.35	17.60	56.06	15.77	0.322
**4**	54.92	16.94	53.51	15.05	0.657
**5**	54.20	18.14	49.14	19.03	0.172

**Table 4:** Perceptions of dentists and non-professionals towards increment level of tilted occlusal plane.

Tilted Occlusal Plane (°)	Non-Professionals		(Dentists)		*p*
	x	SD	x	SD	
**0**	53.27	15.75	52.47	15.45	0.795
**1**	51.16	16.35	52.02	15.37	0.784
**2**	53.06	12.23	52.75	15.25	0.909
**3**	48.02	14.68	45.02	15.56	0.319
**4**	42.82	18.69	37.90	16.83	0.165
**5**	38.09	15.90	32.37	15.33	0.067
